# Succinylation: novel molecular mechanisms and prospects for targeted therapy in liver diseases

**DOI:** 10.3389/fmolb.2026.1768199

**Published:** 2026-02-18

**Authors:** Jie Zhou, Xiaoling Tian, Yifang Zhou, Nianhua Tan, Bin Chen

**Affiliations:** 1 Department of Hepatology, The First Hospital of Hunan University of Chinese Medicine, Changsha, Hunan, China; 2 Department of Traditional Chinese Medicine, School of Health and Medicine, China Three Gorges University, Yichang, Hubei, China; 3 Department of Gastroenterology, Hunan Provincial Hospital of Integrated Traditional Chinese and Western Medicine, Changsha, Hunan, China

**Keywords:** epigenetic, immune, liver disease, metabolism, post-translational modification, succinylation, therapeutic target

## Abstract

Succinylation is a novel post-translational modification involving the attachment of a negatively charged succinyl group to lysine residues, which fundamentally alters the structure and function of substrate proteins. The liver, the metabolic center of the body, is a critical target for succinylation because of its high metabolic activity. Growing evidence suggests that succinylation is a core pathological mechanism that bridges hepatic metabolic dysfunction and disease progression by modulating metabolic enzyme activity, influencing epigenetic status, and remodeling the immune microenvironment. This review aimed to systematically outline the molecular features and regulatory networks of succinylation, dissect its mechanistic roles in major liver diseases, and synthesize current therapeutic strategies, including clinical drug repurposing, small-molecule agents, and traditional Chinese medicinal compounds that target this modification. Collectively, these insights offer a novel theoretical framework and promising therapeutic direction for the management of liver diseases.

## Graphical Abstract

**Figure:**
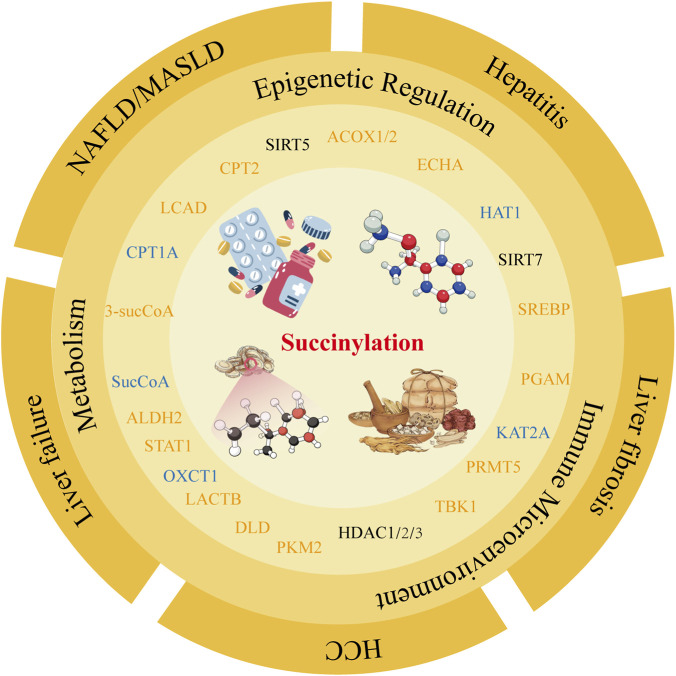

## 1 Introduction

Protein post-translational modifications (PTMs) regulate protein stability, activity, and subcellular localization through covalent modifications and represent a fundamental mechanism underlying protein functional diversity (Zhang et al., 2011; Gao et al., 2020; Lee et al., 2023). Succinylation, an emerging PTM, adds negatively charged succinyl groups to lysine residues, altering protein conformation and charge to modulate physicochemical properties and biological functions. Succinylation plays essential roles in metabolic regulation, signal transduction, and cellular differentiation, establishing itself as a key focus in epigenetics and metabolism research (Zhang H. et al., 2023; Ye and Li, 2022; Amirkasha et al., 2023; Huang et al., 2023). As cellular powerhouses, mitochondria serve as the primary sites of succinylation. This modification directly contributes to mitochondrial homeostasis by regulating the activity of key enzymes involved in metabolic pathways such as the tricarboxylic acid (TCA) cycle and fatty acid oxidation (Yang et al., 2022; Xiao et al., 2021; Hou et al., 2025).

These findings highlight succinylation as a pivotal molecular mechanism linking cellular metabolism to functional regulation. The liver, as the body’s metabolic hub, contains mitochondria-rich cells. Its high metabolic activity provides abundant substrates for succinylation, resulting in higher levels in the liver than in most other tissues (Rardin et al., 2013). Consequently, the liver is highly sensitive to succinylation dysregulation, with impaired succinylation closely associated with the onset and progression of various liver diseases (Liu et al., 2024a).

Recent studies have underscored the critical role of succinylation in maintaining hepatic metabolic homeostasis (Hou et al., 2024). Its importance is increasingly recognized in the development of diverse liver conditions, including fatty liver disease (Wu et al., 2014), viral hepatitis (Ren et al., 2025), and hepatocellular carcinoma (HCC) (Wang Z. et al., 2025). However, existing evidence is largely limited to single disease models or isolated molecular events and lacks a systematic framework to elucidate the central role of succinylation and its mechanistic connections across different liver diseases. This review aimed to systematically characterize the role of succinylation in the pathological processes of multiple liver diseases, beginning with its underlying molecular mechanisms. We propose that succinylation functions as a central regulatory hub integrating liver metabolic disorders, epigenetic dysregulation, and immune microenvironment imbalance. Furthermore, we examine the therapeutic potential and recent advances in targeting succinylation for liver disease treatment to provide new directions and strategies for the precise prevention and management of liver diseases.

## 2 Molecular characteristics and regulatory mechanisms of succinylation

### 2.1 Biochemical properties

Succinylation is the chemical process where a succinyl group (–CO–CH_2_–CH_2_–COO^-^) covalently binds to the ε-amino group of a lysine residue via enzymatic or non-enzymatic pathways, forming an amide bond (Papan et al., 2014). The core biochemical characteristics of this modification are charge inversion and induced steric hindrance. Under physiological conditions, the lysine ε-amino group carries a positive charge (+1), whereas the introduced succinyl group is negatively charged (−1). This charge inversion from +1 to −1 significantly alters the electrostatic landscape of the protein surface, consequently influencing its conformation, enzymatic activity, protein–protein interactions, and subcellular localization (Zhang et al., 2011; Ramazi and Zahiri, 2021). In contrast, acetylation introduces a neutral acetyl group (–CO–CH_3_), which neutralizes the positive charge (from +1 to 0) (Charidemou and Kirmizis, 2024). Furthermore, the succinyl group is structurally longer and bulkier than the acetyl group, generating more pronounced steric hindrance that can disrupt local protein architecture and functional interfaces (Hirschey and Zhao, 2015). Consequently, succinylation exerts a more substantial impact on protein structure and function compared to acetylation, enabling it to act as an efficient metabolic “switch” that rapidly responds to changes in cellular metabolic states.

### 2.2 Regulatory mechanisms of succinylation

#### 2.2.1 Succinyl donors

Succinyl-coenzyme A (SucCoA), the direct substrate for succinylation, determines modification levels through its intracellular concentration, particularly by dominating non-enzymatic pathways (Lancaster et al., 2023; Zhang J. et al., 2022). In highly metabolic tissues, such as the liver, or under specific pathological conditions, abnormal accumulation of SucCoA directly reacts with lysine residues on proteins via non-enzymatic pathways, leading to widespread succinylation (Lancaster et al., 2023; Sadhukhan et al., 2016). Conversely, limiting SucCoA suppresses the succinylation of mitochondrial proteases (Yan et al., 2024). Furthermore, dietary succinate influences protein succinylation levels via non-enzymatic pathways, providing a molecular explanation of environmental and dietary factors that directly regulate cellular functions (Guillon et al., 2022; Ding et al., 2022) (Table 1).

**TABLE 1:** Key enzymes regulating protein succinylation and their roles in liver disease.

Category	Enzyme/Molecule	Subcellular localization	Mechanism of action	Association with liver disease
Succinyl Donor	SucCoA	Primarily mitochondria	Direct succinyl-group donor; its intracellular concentration is a major determinant of global succinylation levels (Lancaster et al., 2023; Zhang J. et al., 2022)	Highly abundant in the liver; its dysregulated accumulation is linked to metabolic disorders
Succinyltransferases	α-KGDHC	Mitochondria	Serves dual roles: A key enzyme generating succinyl-CoA in the TCA cycle; Possesses intrinsic succinyltransferase activity, directly catalyzing protein succinylation to regulate metabolism (Piroli et al., 2023; Hansen and Gibson, 2022)	May drive metabolic liver disease progression by dysregulating core energy metabolism through succinylation; its precise pathogenic role requires further elucidation
	KAT2A	Nucleus/Cytoplasm	Catalyzes succinylation of histone (e.g., H3K79) and non-histone (e.g., PKM2), promoting transcriptional activation and glycolysis (Zhang and Huang, 2024; Qin et al., 2021)	Drives HBV cccDNA transcription and promotes HCC progression
	HAT1	Nucleus	Catalyzes succinylation of histone H3 and the metabolic enzyme PGAM1, linking epigenetic regulation to glycolytic flux (Yang et al., 2021)	Promotes glycolysis and proliferation in HCC
	CPT1A	Mitochondria	Possesses succinyltransferase activity; catalyzes protein succinylation to modulate fatty acid metabolism (Liu et al., 2024b)	Associated with lipid metabolism disorders
	OXCT1	Mitochondria	Catalyzes succinylation of LACTB and PGK1, thereby influencing cellular energy metabolism (Ma et al., 2024; Zhang H. et al., 2025)	Upregulated in HCC; promotes tumor growth
Desuccinylases	SIRT5	Mitochondria	Major mitochondrial desuccinylase; targets key metabolic enzymes (e.g., ECHA, ACOX1, ALDH2) to regulate fatty acid oxidation and mitigate oxidative stress (Tang et al., 2025; Chen et al., 2018; Chiba et al., 2024)	Exerts protective roles in NAFLD and liver failure; its expression is often downregulated in disease
	SIRT7	Nucleus	Catalyzes desuccinylation of histones (e.g., H3K122), modulating gene transcription and viral replication (Yu et al., 2021)	Suppresses HBV transcription; frequently overexpressed in HCC and associated with poor prognosis
	HDAC1/2/3	Nucleus	Function as major histone desuccinylases; regulate transcriptional activity by maintaining low succinylation at gene promoters (Li et al., 2023; Guo et al., 2020)	Participate in the transcriptional regulation of lipogenic genes (e.g., SREBP1c) in NAFLD

#### 2.2.2 Succinyltransferases

Succinyltransferases positively regulate succinylation via enzymatic reactions. Currently identified succinyltransferases exhibit substrate diversity and functional complexities. Importantly, the mitochondrial α-ketoglutarate dehydrogenase complex (α-KGDHC), a central enzyme in the TCA cycle, serves not only as a primary source of SucCoA but also possesses intrinsic succinyltransferase activity. It directly catalyzes protein succinylation, thereby influencing a broad range of cellular metabolic processes (Piroli et al., 2023; Hansen and Gibson, 2022).

Lysine acetyltransferase 2A (KAT2A) exhibits histone and non-histone modification capabilities. It promotes glycolysis in gastric cancer by upregulating succinylation of pyruvate kinase M2 (PKM2) (Zhang and Huang, 2024) and catalyzes succinylation of histone H3 at lysine 79 (H3K79) to enhance viral transcription in hepatitis B (Qin et al., 2021). These findings demonstrate the multifunctionality of KAT2A in diverse pathological states. Histone acetyltransferase 1 (HAT1) catalyzes H3K122 succinylation for epigenetic regulation and targets the K99 site of phosphoglycerate mutase 1 (PGAM1) to promote HCC progression (Yang et al., 2021). This underscores the critical role of HAT1 in linking epigenetics to metabolic reprogramming.

Several other key metabolic enzymes exhibit succinyltransferase activity. Carnitine palmitoyltransferase 1A (CPT1A) and oxoacid CoA-transferase 1 (OXCT1), which are central to fatty acid and ketone body metabolism, respectively, directly catalyze protein succinylation, revealing their non-canonical roles in cellular energy metabolism regulation (Tian et al., 2025; Liu et al., 2024b; Ma et al., 2024; Zhang H. et al., 2025) (Table 1).

#### 2.2.3 Desuccinylases

Desuccinylases negatively regulate succinylation to maintain cellular homeostasis. Key enzymes, including SIRT5, SIRT7, and HDAC1/2/3, form a regulatory system characterized by subcellular compartmentalization. SIRT5, the primary mitochondrial desuccinylase, regulates the succinylation of over 80% of the metabolic enzymes (Rauh et al., 2013; Ke et al., 2025). In SIRT5 knockout mice, succinylation levels increase at 386 lysine sites across 140 metabolic proteins, whereas SIRT5 overexpression effectively reduces protein succinylation levels. These proteins participate extensively in core metabolic pathways such as fatty acid oxidation and the TCA cycle (Rardin et al., 2013; Zhou et al., 2021), indicating that SIRT5 serves as a key regulator of mitochondrial metabolism. SIRT7 primarily resides in the nucleus, where it influences gene expression by regulating histone H3 desuccinylation (Bai et al., 2022; Yu et al., 2021). HDAC1/2/3 are major histone desuccinylases. The inhibition of their activity results in a substantial increase in histone succinylation within promoter regions, thereby influencing the transcription of downstream genes (Li et al., 2023). This observation underscores their essential role in epigenetic regulation (Table 1).

**FIGURE 1:**
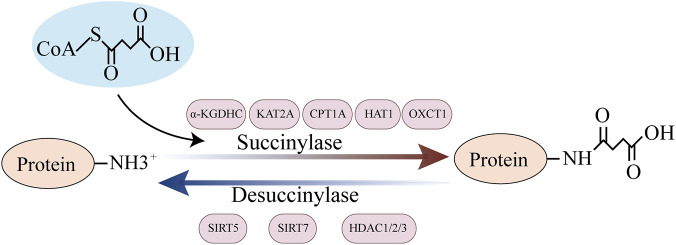
Regulatory mechanisms of succinylation. The mechanisms regulating succinylation can be classified into two categories: (i) non-enzymatic pathways, where SucCoA promotes the succinylation of proteins; and (ii) enzymatic pathways, where succinylases KAT2A, HAT1, CPT1A, and OXCT1 positively regulate succinylation, while desuccinylases SIRT5, SIRT7, and HDAC1/2/3 negatively regulate succinylation.

Succinylation is a highly complex and dynamically balanced regulatory mechanism. Its functional execution relies on synergistic interactions between modifying enzymes, metabolic substrates, and effector proteins (Figure 1). Succinylation is involved in the pathological processes of liver disease at multiple levels by regulating metabolic pathways, epigenetics, and signal transduction.

### 2.3 Crosstalk between succinylation and other PTMs

**FIGURE 2:**
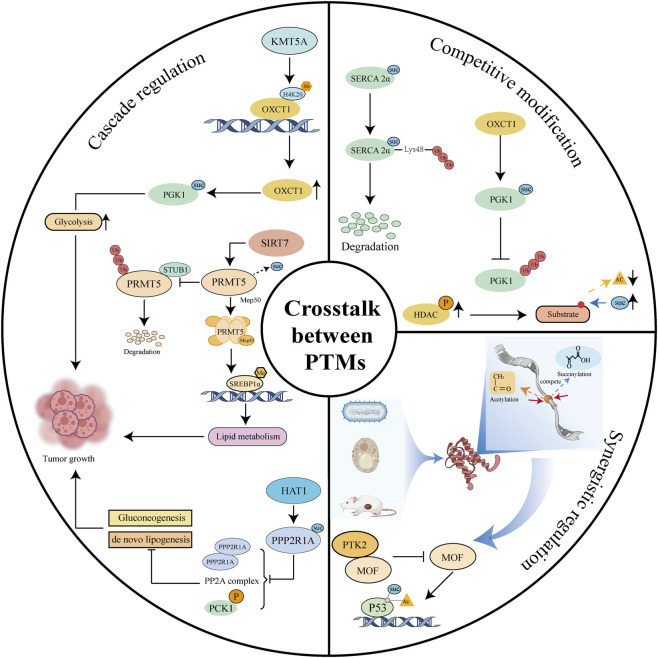
Crosstalk between succinylation and other PTMs.

The precise regulation of protein function relies on a complex network of multiple PTMs. As a modification that is highly sensitive to the cellular metabolic state, succinylation engages in extensive crosstalk with other major PTMs such as acetylation, ubiquitination, methylation, and phosphorylation. These intricate interactions expand the dimensions of protein functional regulation and provide new insights into integrated regulatory mechanisms that link cellular metabolism, epigenetics, and the immune microenvironment (Figure 2).

#### 2.3.1 Cascade regulation mechanisms

Cascade regulation is a precise control pattern in which different PTMs act sequentially according to specific temporal sequences. Lysine methyltransferase 5 A (KMT5A) promotes OXCT1 expression by mediating histone methylation, which in turn enhances succinylation of the PGK1 protein. This methylation–succinylation cascade synergistically promotes aerobic glycolysis and immune evasion in the tumor microenvironment (Zhang H. et al., 2025). Furthermore, SIRT7 desuccinylates protein arginine methyltransferase 5 (PRMT5) at K387, which blocks STIP1 homology and U-box containing protein 1 (STUB1) binding and inhibits PRMT5 ubiquitination and degradation. Stabilized PRMT5 forms complexes with methylosome protein 50 (Mep50) and methylates sterol regulatory element-binding protein 1a (SREBP1a), thereby reprogramming lipid metabolism and fueling tumor progression (Yuan et al., 2022). A similar pathway occurs in hepatocellular carcinoma (HCC). HAT1 catalyzes the succinylation of protein phosphatase 2 scaffold subunit alpha (PPP2R1A) at K541, impairing the assembly of the PP2A holoenzyme and blocking its interaction with phosphoenolpyruvate carboxykinase 1 (PCK1), thereby preventing PCK1 phosphorylation at S90. This remodels glucose metabolism and promotes tumor growth (Yang G. et al., 2025). These cascading regulatory pathways reveal the central role of succinylation as a hub connecting diverse PTM networks.

#### 2.3.2 Competitive modification mechanisms

Competitive modifications, in which different PTMs compete for the same lysine residue, constitute a fundamental aspect of PTM networks. Systems biology studies have indicated extensive site overlap between succinylation and acetylation during evolution. Cross-species analysis has revealed that approximately 37.3% of succinylation sites overlapped with acetylation sites in bacterial, yeast, and mouse livers, demonstrating the universality of the competitive relationship between the two modifications (Weinert et al., 2013). This phenomenon was further validated in *Vibrio alginolyticus*, in which 1,005 of 2,082 succinylation sites simultaneously exhibited acetylation, providing direct site-specific evidence of modification competition (Zeng et al., 2020). Moreover, in an alcohol-induced mitochondrial protein acylation model, acetylation and succinylation at the same lysine residue showed a clear inverse relationship, where enhanced acetylation was correlated with weakened succinylation, intuitively demonstrating dynamic competition between the two modifications (Ali et al., 2019). At the molecular level, regulation of the key tumor suppressor p53 provides a paradigm for understanding competitive modifications. Lysine acetyltransferase 8 (KAT8) can mediate both acetylation and succinylation at the p53 K120 site. Conversely, protein tyrosine kinase 2 (PTK2) competitively binds to KAT8, simultaneously inhibiting acetylation and succinylation at this site, thereby precisely regulating p53 transcriptional activity (Wang Y. et al., 2025). Collectively, these studies from systems-level to molecular mechanisms have established a theoretical framework for competitive succinylation modifications, laying a crucial foundation for deepening our understanding of state transitions in protein function.

#### 2.3.3 Synergistic regulatory networks

Synergistic regulation reveals complex patterns in which distinct PTMs collaborate to jointly modulate protein function. Succinylation of sarcoplasmic/endoplasmic reticulum calcium ATPase 2a (SERCA2a) K352 enhances its degradation by promoting K48-linked ubiquitination (Yang N. et al., 2025). Conversely, OXCT1-mediated succinylation at PGK1 K146 significantly enhances protein stability by inhibiting ubiquitination (Zhang H. et al., 2025), demonstrating that succinylation can either promote or antagonize ubiquitin-dependent turnover. In non-small cell lung cancer, phosphorylation of HDAC1 enhances its deacetylase activity, which exposes lysine residues and promotes mitochondrial protein succinylation, thereby driving metabolic reprogramming (Guo et al., 2024). Clinical samples further revealed a significant positive correlation between HDAC phosphorylation and mitochondrial succinylation in tumors, highlighting phosphorylation as a key coordinator of the acetylation–succinylation balance (Guo et al., 2024). These findings establish a positive feedback loop between phosphorylation and succinylation and provide crucial insights into the synergistic regulation of PTM networks.

The crosstalk mechanisms form a sophisticated multi-layered network. Deciphering the cascade, competitive, and synergistic interactions within this network will not only clarify the pathogenesis of complex diseases, but also provide a theoretical foundation to develop multi-target therapeutic strategies.

## 3 Molecular mechanisms of succinylation in the progression of liver disease

### 3.1 Non-alcoholic fatty liver disease (NAFLD) and metabolic dysfunction-associated steatotic liver disease (MASLD)

**FIGURE 3:**
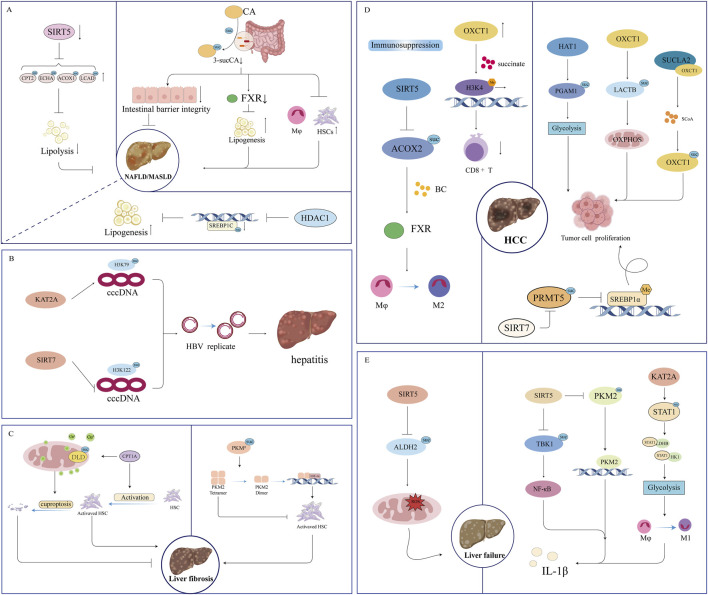
Molecular mechanisms of succinylation in the progression of liver disease. **(A)** NAFLD/MASLD; **(B)** hepatitis; **(C)** liver fibrosis; **(D)** HCC; **(E)** liver failure.

NAFLD, recently redefined as MASLD, is pathologically characterized by the severe dysregulation of hepatic lipid metabolism (Rong et al., 2022; Zhang and Brandman, 2025). Succinylation plays a central role in metabolic dysregulation during NAFLD/MASLD by regulating the activity of key metabolic enzymes in hepatocytes (Cheng et al., 2016) (Figure 3).

#### 3.1.1 Reprogramming of lipid metabolism

SREBP1 regulates hepatic lipogenesis (Bian et al., 2025). HDAC1 maintains low succinylation levels in the *SREBP1c* promoter, enhancing its transcriptional activity and driving the expression of downstream lipogenic genes (Guo et al., 2020). Impaired fatty acid oxidation pathways directly cause lipid accumulation in hepatocytes. Carnitine palmitoyltransferase 2 (CPT2) serves as a crucial enzyme that facilitates fatty acid oxidation. Succinylation of CPT2 at K424 alters the conformation of its active site and directly inhibits its enzymatic activity, impairing the entry of long-chain fatty acids into mitochondria, leading to fatty acid oxidation disorders and accumulation of acyl intermediates (Wu et al., 2024; Li J. et al., 2024). Desuccinylase SIRT5 plays a central role in maintaining the balance of lipid metabolism by regulating desuccinylation of multiple key fatty acid oxidation enzymes, thereby forming a metabolic cascade regulatory mechanism. Specifically, SIRT5-mediated desuccinylation of the trifunctional enzyme subunit alpha (ECHA) at K540 enhances its stability and expression, promoting fatty acid β-oxidation and reducing hepatic steatosis (Tang et al., 2025).

SIRT5 also regulates the activity of other oxidation enzymes, including acyl-CoA oxidase 1 (ACOX1) and long-chain acyl-CoA dehydrogenase, thereby fine-tuning overall fatty acid oxidation efficiency (Chen et al., 2018; Chiba et al., 2024). Clinical studies have further confirmed that SIRT5 expression levels in liver tissue are significantly reduced in patients with NAFLD and are negatively correlated with disease severity (Wu et al., 2014). These findings reveal the pivotal regulatory role of SIRT5 in NAFLD/MASLD progression and its clinical translational value as a potential therapeutic target.

#### 3.1.2 Gut–microbiota–derived succinylated metabolites and the gut–liver axis

Succinylation of bile acid molecules by the gut microbiota represents a critical pathophysiological pathway that influences MASLD development. The representative bacterial bile acid 3-succinoylcholic acid (3-sucCA) is derived from the host cholic acid through microbial catalysis by introducing a succinyl group at its 3-hydroxy position (Chen R. et al., 2025). Its levels are markedly decreased in MASLD patients and animal models and are inversely correlated with disease severity (Nie et al., 2024). The protective effects of 3-sucCA involve multiple synergistic mechanisms: (i) improving intestinal barrier function by regulating gut microbiota (e.g., increasing *Akkermansia muciniphila*) and enhancing tight junction protein expression in intestinal epithelial cells, thereby reducing endotoxin translocation; (ii) activating the farnesoid X receptor (FXR) in the liver and intestine to regulate bile acid synthesis and lipid-metabolism genes, suppressing *de novo* lipogenesis; and (iii) directly inhibiting the activation of hepatic macrophages and the proliferation of hepatic stellate cells (HSCs), thereby mitigating inflammatory responses and the progression of fibrosis (Nie et al., 2024; Liu et al., 2024c). These findings reveal the endogenous protective role of bacterial succinyl-metabolites in MASLD/NAFLD and provide novel targets and translational directions for gut microbiota-based intervention strategies.

### 3.2 Viral hepatitis

Hepatitis B virus (HBV) infection is a leading cause of viral hepatitis in China and a major driver of liver fibrosis, cirrhosis, and HCC. HBV covalently closed circular DNA (cccDNA) forms minichromosomes within infected hepatocyte nuclei, serving as a template for viral RNA transcription, and constituting a critical factor in persistent infection and treatment resistance (Ren et al., 2025). The transcriptional activity of cccDNA is regulated by histone modifications, of which succinylation has emerged as a critical epigenetic mechanism and a potential therapeutic target (Figure 3).

#### 3.2.1 Activation of viral transcription

Viruses hijack host epigenetic machinery to sustain replication. Host succinyltransferase KAT2A directly interacts with the HBV core protein (HBc) and recruits it to cccDNA minichromosomes, where it catalyzes the succinylation of H3K79. This modification significantly enhances cccDNA transcriptional activity and viral replication (Qin et al., 2021).

#### 3.2.2 Antiviral innate immune responses

In contrast, SIRT7 inhibits viral transcription by catalyzing histone deacetylation. Succinylated H3K122 accumulates in transcriptionally active cccDNA and correlates positively with transcription levels. SIRT7 binds to HBc and is recruited to cccDNA, where it catalyzes H3K122 desuccinylation, thereby repressing transcription (Yu et al., 2021). This reveals an innate immune strategy in which the host combats viral infections by regulating epigenetic mechanisms, offering new molecular insights into innate immune responses.

KAT2A and SIRT7 form functional antagonists during viral transcription by regulating the succinylation of HBV cccDNA. Therefore, inhibiting KAT2A or enhancing SIRT7 activity to maintain the “write-erase” dynamic equilibrium provides new targets to develop antiviral strategies based on epigenetic mechanisms.

### 3.3 Liver fibrosis and cirrhosis

Liver fibrosis is a reparative response to chronic liver injury, characterized by excessive extracellular matrix deposition. Cirrhosis constitutes the terminal stage of fibrosis, manifesting as structural destruction and a functional decrease of the liver (Ginès et al., 2021; Somnay et al., 2024). The pathogenesis of liver fibrosis and cirrhosis is complex and involves interactions between multiple cell types, signaling pathways, and molecular events (Horn and Tacke, 2024; Gilgenkrantz et al., 2021). Analyzing the progression of this disease from the perspective of succinylation aids in elucidating the underlying pathophysiological mechanisms and provides a crucial theoretical foundation for the development of novel targeted therapeutic strategies (Figure 3).

#### 3.3.1 Dual regulation of HSC activation and clearance

HSC activation is the core pathological mechanism involved in the progression of liver fibrosis (Kisseleva and Brenner, 2021). Clinical sample analysis reveals significantly elevated expression of succinyltransferase CPT1A in the HSCs of patients with fibrosis, which positively correlate with the severity of tissue fibrosis (Fondevila et al., 2022). Further experimental studies confirm that downregulation of CPT1A expression effectively inhibits HSC activation and attenuates liver fibrosis (Fondevila et al., 2022; Li Z. et al., 2024). However, within a microenvironment characterized by copper ion homeostasis imbalance, CPT1A catalyzes the succinylation of dihydrolipamide dehydrogenase (DLD), triggering cuproptosis and selectively eliminating activated HSCs, thereby exerting antifibrotic effects (Tian et al., 2025). This dual role suggests that CPT1A function is context-dependent and is shaped by metabolic and microenvironmental states.

#### 3.3.2 Synergistic metabolic and epigenetic drivers

Succinylation of the glycolytic enzyme PKM2 promotes its transition from the active tetrameric form to the less active dimeric form and facilitates dimer nuclear translocation. In the nucleus, dimeric PKM2 forms a transcriptional complex with hypoxia-inducible factor-1α (HIF-1α) to upregulate genes involved in glycolysis and fibrogenesis (Zeng et al., 2024; Zheng et al., 2021; Yang X. Z. et al., 2025). In fibrotic livers, PKM2 is specifically upregulated in activated HSCs, which release PKM2-enriched exosomes. These exosomes induce glycolytic reprogramming in neighboring quiescent HSCs, macrophages, and endothelial cells, creating a positive feedback loop that accelerates fibrosis (Zheng et al., 2020; Wan et al., 2019). Promoting PKM2 tetramerization, in contrast, suppresses HSC activation and glycolysis (Zheng et al., 2020). Although direct evidence linking PKM2 succinylation to its profibrotic functions in HSCs remains to be fully established, the modification-driven shift toward the dimeric/nuclear form provides a plausible mechanistic link between metabolic sensing and fibrogenic transcription. Thus, PKM2 constitutes a critical node integrating metabolic state, epigenetic regulation, and intercellular communication in fibrosis, and modulating its succinylation represents a rational strategy for therapeutic intervention.

### 3.4 HCC

HCC development is accompanied by significant metabolic reprogramming that influences the tumor microenvironment (Lin et al., 2024; Yang et al., 2025d). Succinylation exerts multidimensional regulatory effects on HCC by mediating metabolic reprogramming and reshaping the tumor immune microenvironment, thereby promoting malignant proliferation, invasion, metastasis, and immune evasion (Figure 3).

#### 3.4.1 Reprogramming of energy metabolism

HAT1 is abnormally overexpressed in HCC and catalyzes succinylation of PGAM1 at K99, enhancing its activity, accelerating glycolysis, and promoting tumor growth (Yang et al., 2021). Beyond glycolysis, abnormal ketone body metabolism provides energy support for tumor cell growth (Huang et al., 2016; Tsuru et al., 2024). Ketolytic enzyme OXCT1 is upregulated in HCC (Huang et al., 2016). By interacting with the β-subunit of ADP-forming succinyl-CoA ligase (SUCLA2), it generates succinyl-CoA that drives its own succinylation at K421, enhancing ketone catabolism and proliferation (Guo et al., 2025). OXCT1 also acts as a succinyltransferase, modifying the mitochondrial protease serine β-lactamase-like protein (LACTB) at K284. Succinylation inhibits LACTB protease activity and promotes oxidative phosphorylation and tumor growth (Ma et al., 2024). Clinically, LACTB K284 succinylation correlates positively with tumor size and stage, and negatively with prognosis, suggesting its utility as a prognostic biomarker and therapeutic target (Ma et al., 2024).

#### 3.4.2 Remodeling of the tumor immune microenvironment

Succinylation regulates tumor cell metabolism and actively participates in the remodeling of the tumor immune microenvironment, influencing immune surveillance and evasion. SIRT5 deficiency elevates the succinylation of ACOX2, disrupting bile acid metabolism and promoting M2-like polarization of tumor-associated macrophages via FXR signaling, thereby facilitating immune escape (Sun et al., 2022). Clinical studies have confirmed that high OXCT1 expression in the tumor-associated macrophages of patients with HCC correlates closely with a poor prognosis. OXCT1 overexpression drives abnormal succinate accumulation, which, in turn, activates the transcription program of Arg*1* by upregulating H3K4 trimethylation (H3K4me3), leading to CD8 + T-cell exhaustion (Zhu et al., 2024). KAT2A, acting as both an acetyltransferase and succinyltransferase, acetylates H3K9 in the *TGFB1* promoter to activate TGF-β expression, suppressing T-cell function and promoting immune evasion (Yin et al., 2025). However, the mechanism underlying KAT2A-mediated succinylation in HCC immune regulation remains unclear, and most studies have focused on its acetyltransferase function. Future studies should explore the specific role of KAT2A-mediated succinylation in the dynamic evolution of HCC immune microenvironment.

#### 3.4.3 Desuccinylation maintains tumor stemness

SIRT7, a nuclear-localized desuccinylase, is highly expressed in HCC tissues. Its expression levels correlate positively with tumor stage, vascular invasion, and poor prognosis, making it a key driver of HCC progression (Bai et al., 2022; Gu et al., 2024; Yanai et al., 2020; Zhao et al., 2019). SIRT7 desuccinylation of PRMT5 at K387 enhances methyltransferase activity. This promotes the arginine methylation of SREBP1a, upregulates lipogenic genes, and supports rapid tumor proliferation and migration (Yuan et al., 2022). High SIRT7 expression is significantly associated with resistance to transarterial chemoembolization (TACE) therapy, suggesting its potential role in mediating treatment resistance (Zhao et al., 2019). Given the pivotal role of SIRT7 in the pathological progression of HCC and treatment resistance, an in-depth exploration of targeted therapeutic strategies against SIRT7 has significant clinical value.

### 3.5 Liver failure

Liver failure is a clinically critical syndrome characterized by the rapid deterioration of liver function, multi-organ dysfunction, and extremely high mortality. Its pathophysiological mechanisms involve multiple pathways, including systemic inflammatory response, immune metabolic dysregulation, and oxidative stress, ultimately leading to mitochondrial dysfunction and disruption of hepatic microenvironment homeostasis (Luo et al., 2023). Succinylation, a functional metabolic regulatory hub, plays a central role in mitochondrial dysfunction and inflammatory immune regulation during liver failure (Figure 3).

#### 3.5.1 Mitochondrial dysfunction

Mitochondrial dysfunction is a core pathological component of liver failure. In acetaminophen-induced acute liver injury models, SIRT5 expression is downregulated, leading to increased succinylation of aldehyde dehydrogenase 2 (ALDH2) and the inhibition of its activity (Yu et al., 2024). This results in toxic aldehyde accumulation, glutathione depletion, and excessive reactive oxygen species (ROS) production, ultimately exacerbating hepatocyte death via mitochondrial pathways (Yu et al., 2024; Duan et al., 2025). Therefore, targeting the SIRT5–ALDH2 axis to regulate succinylation may represent a novel strategy to alleviate mitochondrial oxidative stress damage and delay the progression of liver failure.

#### 3.5.2 Immune activation and inflammatory cascades

Liver failure is frequently accompanied by intense local and systemic inflammatory responses, which are key factors that contribute to tissue injury and multiple organ failure. Succinylation regulates inflammatory and immune responses via multiple mechanisms. For example, SIRT5-mediated desuccinylation of TANK-binding kinase 1 (TBK1) at K137 inhibits its activation and downstream pro-inflammatory signaling pathways such as NF-κB/IRF, thereby exerting anti-inflammatory effects (Gorbunova and Seluanov, 2025). Conversely, KAT2A catalyzes the succinylation of the transcription factor signal transducer and activator of transcription 1 (STAT1) at K665, promoting its binding to the promoters of glycolytic genes such as hexokinase 1 (*HK1*) and lactate dehydrogenase B (*LDHB*). This drives metabolic reprogramming and M1 macrophage polarization, thereby amplifying inflammation (Li et al., 2025). Furthermore, SIRT5 desuccinylates PKM2 to anchor it in the cytoplasm, whereas SIRT5 deficiency promotes nuclear translocation of PKM2, where it acts as a coactivator to enhance expression of inflammatory mediators like IL-1β (Wang et al., 2017). Gamma-aminobutyric acid (GABA) signaling also enhances oxidative phosphorylation by reducing overall mitochondrial protein succinylation levels, thereby inhibiting inflammasome activation and IL-1β production (Fu et al., 2022). These findings collectively underscore the pivotal role of succinylation in inflammatory and immune networks.

In summary, dysregulated succinylation constitutes a common pathological basis for multiple liver diseases including NAFLD/MASLD, viral hepatitis, liver fibrosis, HCC, and liver failure. This modification forms a systemic regulatory mechanism that links cellular metabolism to disease progression by modulating metabolic enzymes, reshaping epigenetic states, and influencing the immune microenvironment. The widespread downregulation or dysfunction of desuccinylases appears to be a shared molecular basis for impaired hepatic adaptation to metabolic, oxidative, and inflammatory stresses. Although the preclinical evidence is compelling, the translational potential of succinylation awaits validation through clinical studies (Table 2).

**TABLE 2:** Roles of succinylation in liver diseases.

Disease	Succinylation event	Key effector (s)	Pathological consequence	References
NAFLD/MASLD	↑ CPT2 K424, ECHA K540 succinylation	SIRT5↓	Inhibits fatty acid β-oxidation, promoting hepatic lipid accumulation	Wu et al. (2024), Li J. et al. (2024), Tang et al. (2025)
	↓ Succinylation at *SREBP1c* promoter	HDAC1	Enhances transcriptional activity of SREBP1c, driving *de novo* lipogenesis	Bian et al. (2025), Guo et al. (2020)
	↓ 3-SucCA levels	Gut microbiota	Impairs FXR-mediated hepatoprotection, including barrier integrity, anti-inflammation, and lipid suppression	Chen R. et al. (2025), Nie et al. (2024), Liu et al. (2024c)
Viral Hepatitis	↑ H3K79 succinylation on cccDNA	KAT2A	Activates viral transcription, sustaining persistent HBV infection	Qin et al. (2021)
	↓ H3K122 desuccinylation on cccDNA	SIRT7↓	Compromises innate antiviral response, facilitating viral persistence	Yu et al. (2021)
Liver Fibrosis/Cirrhosis	↑ DLD succinylation	CPT1A	Induces cuproptosis in activated HSCs, exerting context-dependent antifibrotic effects	Tian et al. (2025)
	↑ PKM2 succinylation	—	Promotes HSC activation, metabolic reprogramming, and fibrogenic signaling	Zeng et al. (2024), Zheng et al. (2021), Yang N. et al. (2025)
HCC	↑ PGAM1 K99 succinylation	HAT1	Enhances glycolysis to fuel tumor growth	Yang et al. (2021)
	↑ LACTB K284 succinylation	OXCT1	Suppresses mitochondrial protease activity, promoting oxidative phosphorylation	Ma et al. (2024)
	↑ ACOX2 succinylation	SIRT5↓	Disrupts bile acid metabolism, fostering an immunosuppressive M2-like TAM phenotype	Sun et al. (2022)
	↑ Succinate accumulation → H3K4me3 at Arg*1*	OXCT1	Drives Arg*1* expression in macrophages, leading to CD8^+^ T-cell exhaustion	Zhu et al. (2024)
	↑ PRMT5 K387 desuccinylation	SIRT7	Enhances PRMT5 methyltransferase activity, upregulating lipogenesis and tumor progression	Yuan et al. (2022)
Liver Failure	↑ ALDH2 succinylation	SIRT5↓	Impairs detoxification, leading to toxic aldehyde accumulation, oxidative stress, and hepatocyte death	Yu et al. (2024), Duan et al. (2025)
	↓ TBK1 desuccinylation ↑ STAT1 K665 succinylation ↑ PKM2 succinylation/nuclear translocation	SIRT5↓ KAT2A	Activates pro-inflammatory signaling (e.g., NF-κB/IRF, STAT1), promotes M1 macrophage polarization, and amplifies inflammatory cascades	Gorbunova and Seluanov (2025), Li et al. (2025), Wang et al. (2017)

## 4 Drug interventions and therapies targeting succinylation

### 4.1 Succinylation regulatory potential of commonly used clinical drugs

Multiple commonly used clinical drugs exert their therapeutic effects by modulating succinylation, thereby offering new perspectives for the treatment of liver diseases. Beyond its classic immunomodulatory effects, the frontline antiviral drug IFN-α downregulates KAT2A expression via the JAK-STAT pathway, thereby reducing H3K79 succinylation on cccDNA and inhibiting HBV transcription (Yuan et al., 2020). This finding provides a novel mechanistic perspective for IFN-α′s antiviral action and validates KAT2A as a potential therapeutic target. Metformin hydrochloride activates the AMPK-SIRT5 axis, promoting SIRT5-mediated desuccinylation of ECHA. This improves glucose and lipid metabolism and restores mitochondrial function, revealing a new molecular basis for its use in NAFLD/MASLD (Tang et al., 2025). Glibenclamide inhibits the succinyltransferase activity of CPT1A, thereby blocking its mediation of mitochondrial fission factor (MFF) succinylation, which subsequently affects the formation of mitochondria-associated membranes and the activation of the lipogenic transcription factor SREBP1 (Zhu Y. et al., 2025). In HCC, glibenclamide activates the JNK pathway, increases intracellular ROS levels, induces apoptosis, and suppresses tumor progression (Yan et al., 2017). Additionally, it ameliorates drug-induced liver injury by inhibiting hepatic bile acid secretion (Le Vee et al., 2022). These findings collectively demonstrate the potential of glibenclamide in the treatment of liver diseases through the modulation of the succinylation pathway. Aspirin competitively binds to the p65 subunit of NF-κB, inhibiting its nuclear translocation. This blocks the transcriptional activation of the HAT1 promoter, downregulates HAT1 expression, and reduces K99 succinylation levels of PGAM1. Ultimately, it exerts antitumor effects by inhibiting the Warburg effect (Wang et al., 2023). Lidocaine promotes SIRT5-mediated desuccinylation of histone H2B-like proteins, demonstrating its therapeutic potential for the targeted treatment of HCC (Chen X. et al., 2024). Furthermore, acetylhydroxylysine, a clinically used urinary tract infection treatment and potent OXCT1 inhibitor, demonstrates significant synergistic antitumor effects when combined with lenvatinib in a mouse liver tumor model (Guo et al., 2025). These studies reveal novel mechanisms by which existing drugs regulate succinylation modification, providing theoretical support for repurposing old drugs. The findings also validate the feasibility of targeting key succinylation enzymes and lay the foundation for developing combination therapies based on complementary mechanisms of action.

### 4.2 Small-molecule agonists and inhibitors

Significant progress has been made in the development of small molecules that target key enzymes involved in succinylation. Multiple compounds have demonstrated clear therapeutic potential. The SIRT7 inhibitors 2800Z and 40569Z specifically bind to the active site of the enzyme, effectively inhibiting its desuccinylation activity. Preclinical studies demonstrate synergistic antitumor effects when combined with sorafenib (Zhang et al., 2021; Zhang C. et al., 2023). The HDAC2-specific inhibitor CAY10683 mitigates intestinal injury in acute liver failure by blocking the mitochondrial apoptotic pathway and reducing the expression of the pro-apoptotic protein Bax expression (Liu et al., 2019). Compound D574-0246 effectively inhibited the dual enzyme activity of OXCT1, reduced LACTB succinylation in a dose-dependent manner, and suppressed tumor growth (Liu H. et al., 2025). Naturally derived inhibitors exhibit several unique advantages. Aurora fibroblastin A specifically binds to the Gln299 and Asp305 residues of SIRT7, inhibiting its enzymatic activity. This promotes succinylation and subsequent proteasomal degradation of PRMT5, thereby activating the cGAS-STING pathway and inducing HSC senescence (Wang J. et al., 2025). Hydroxytyrosol exerts hepatoprotective effects by inhibiting HDAC1/2 activity, activating hepatic autophagy, and alleviating oxidative stress and inflammation (Fan et al., 2024). Furthermore, compounds such as astragaloside IV (Zhu and Lu, 2024), crocin (Chen H. et al., 2025), and paeoniflorin (Chen R. et al., 2024) play crucial roles in treating tumors and inflammatory diseases by downregulating KAT2A expression and inhibiting succinylation of its substrate protein.

Studies on small-molecule agonists have primarily focused on SIRT5 activation. 2,3,5,4′-Tetrahydroxystyrene-2-O-β-d-glucoside (TSG) enhances the stability of SIRT5 mRNA by strengthening the interaction with the RNA-binding protein SRSF2, thereby increasing its protein expression levels. This upregulation promotes CPT1A activity and ameliorates hepatic lipid metabolism disorders (Zhang S. et al., 2022). Puerarin activates the AMPK pathway to upregulate SIRT5 expression, specifically catalyzing desuccinylation of the key site K385 on ALDH2. This enhances ALDH2 enzyme activity, promotes the clearance of aldehyde substances, and effectively alleviates oxidative stress damage (Yu et al., 2024). Natural products, such as quercetin (Chang et al., 2024; Chang et al., 2021), resveratrol (Yang et al., 2025e; Zhang L. et al., 2025), and ligustrazine (Shi et al., 2025), directly activate SIRT5 to promote the desuccinylation of substrates, such as isocitrate dehydrogenase 2 (IDH2) and dual-specificity phosphatase 1 (DUSP1), thereby maintaining mitochondrial energy homeostasis. In addition to natural products, the synthetic small-molecule agonist, MC3138, selectively activates SIRT5 to regulate glutamine metabolism. Its combination with gemcitabine or the phosphate chelator lanthanum acetate has synergistic antitumor effects, offering new directions for developing SIRT5-targeted combination therapies (Hu et al., 2021; Barreca et al., 2023).

These studies validate the feasibility of succinylation-regulating enzymes as therapeutic targets and lay a solid foundation for the development of novel targeted treatment strategies. With deeper elucidation and optimization of compound mechanisms, small-molecule drugs targeting succinylation modifications hold promise as novel therapeutic approaches for liver diseases. Future studies should focus on enhancing compound selectivity and specificity to advance clinical translation.

### 4.3 Multi-target modulators

Certain natural bioactive compounds exert therapeutic effects by coordinately regulating multiple key nodes within the succinylation network, reflecting the characteristics of multi-target intervention strategies. Allicin and its metabolites are representative of such modulators, exhibiting a pronounced dose-dependent bidirectional regulatory profile (Liu et al., 2023; Li W. et al., 2022). Moderate allicin intake inhibits SIRT5 activity, upregulates uncoupling protein-1 (UCP1) succinylation in brown adipocytes, accelerates energy expenditure, suppresses lipid accumulation, and improves hepatic steatosis. Excessive allicin further inhibits SIRT5-mediated desuccinylation, leading to excessive UCP1 succinylation, inducing mitochondrial autophagy and ultimately causing morphological abnormalities and energy metabolism disorders (Zhang et al., 2020). Its active metabolite diallyl trisulfide (DATs) regulates RAB18 phase separation, upregulates CPT1A expression, promotes DLD succinylation, and induces cuproptosis in HSC (Tian et al., 2025). Thus, through the synergistic actions of the parent compound and its metabolites, allicin achieves dual regulation of the SIRT5–UCP1 pathway and the CPT1A–DLD pathway. Curcumin exerts hepatoprotective effects through multiple pathways. It inhibits ferroptosis by upregulating SIRT5-mediated desuccinylation of acyl-CoA synthetase long-chain family member 4 (ACSL4) and modulates lipid metabolism by suppressing CPT1A expression, demonstrating multi-target regulatory properties (Yashmi et al., 2024; Li R. et al., 2022; Xu et al., 2025).

The primary advantage of such multi-target modulators lies in their ability to exert more systematic and balanced regulation of the succinylation network, rather than excessive intervention at a single node, making them potentially more suitable for treating complex network-dysregulation diseases such as metabolic dysfunction-associated steatotic liver disease. However, their multi-target nature also presents challenges in elucidating mechanisms of action and optimizing clinical dosing.

### 4.4 Traditional Chinese medicine formulas

Traditional Chinese Medicine (TCM) formulas exhibit unique advantages in regulating the dynamic balance of protein succinylation through multi-component synergy. Tianyang Wan (Tianyang Pill), composed of six herbs, Morinda root (*Morindae officinalis* Radix), Cistanche (*Cistanches* Herba), Cynomorium (*Cynomorii* Herba), processed Rehmannia root (*Rehmanniae* Radix Praeparata), Cornus fruit (*Corni* Fructus), and Chinese yam (*Dioscoreae* Rhizoma), can reverse the abnormally elevated global protein succinylation levels in HCC. Integrated proteomic and lysine-succinylomic analyses have demonstrated that Tianyang Wan specifically downregulates succinylation levels of key glycolytic enzymes, including PKM2, fructose-bisphosphate aldolase A (ALDOA), and LDHA, thereby suppressing the glycolytic process in cancer cells and exerting an antitumor effect (Lu, 2023). This effect suggests that the formulation exerts broad influence over the enzymatic machinery governing succinylation. Huanglian Wendan Tang (Huanglian Wendan Decoction), which contains active compounds such as quercetin and berberine, ameliorates NAFLD by synergistically modulating the CPT1A/PPARα signaling pathway to improve lipid metabolism (Zhu J. et al., 2025). Furthermore, Yiqu Gubiao Wan (Yiqu Gubiao Pill) enhances mitochondrial function and related coenzyme synthesis through upregulation of SIRT5 expression, offering a novel therapeutic approach for metabolic liver diseases (Meng, 2021) (Table 3).

**TABLE 3:** Therapeutic strategies targeting protein succinylation.

Therapeutic category	Agent/Compound	Primary target (s)	Key mechanism and Pharmacological effect	Current stage	References
Clinical Drug Repurposing	IFN-α	KAT2A	Downregulates KAT2A expression, reduces H3K79 succinylation on HBV cccDNA, and inhibits viral transcription	Preclinical	Yuan et al. (2020)
	Metformin	AMPK/SIRT5/ECHA	Activates AMPK-SIRT5 signaling, promotes ECHA desuccinylation, and improves glucose/lipid metabolism	Preclinical	Tang et al. (2025)
	Glibenclamide	CPT1A/MFF	Inhibits CPT1A succinyltransferase activity, blocks MFF succinylation, and improves mitochondrial function	Preclinical	Zhu Y et al. (2025), Yan et al. (2017), Le Vee et al. (2022)
	Aspirin	NF-κB/HAT1 pathway	Inhibits NF-κB nuclear translocation, downregulates HAT1, and reduces PGAM1 K99 succinylation	Preclinical	Wang et al. (2023)
	Acetylhydroxylysine	OXCT1	Potently inhibits OXCT1 activity; exhibits synergistic antitumor effects with lenvatinib	Preclinical	Guo et al. (2025)
Synthetic Small-Molecule Modulators	Inhibitors				
	2800Z/40569Z	SIRT7	Specifically inhibit SIRT7 desuccinylase activity; synergize with sorafenib to exert antitumor effects	Preclinical	Zhang et al. (2021), Zhang C. et al. (2023)
	CAY10683	HDAC2	Specific HDAC2 inhibitor; blocks the mitochondrial apoptosis pathway (e.g., reduces Bax)	Preclinical	Liu et al. (2019)
	D574-0246	OXCT1	Inhibits the dual enzyme activity of OXCT1, reducing succinyl-CoA levels and LACTB succinylation	Preclinical	Liu R et al. (2025)
	Agonist				
	MC3138	SIRT5	Selective SIRT5 agonist; modulates glutamine metabolism	Preclinical	Hu et al. (2021), Barreca et al. (2023)
Natural Product Derivatives	Aurorafibroblastin A	SIRT7/PRMT5	Binds and inhibits SIRT7, promotes succinylation and degradation of PRMT5, and activates the cGAS-STING pathway	Preclinical	Wang Z. et al. (2025)
	Hydroxytyrosol	HDAC1/2	Inhibits HDAC1/2, activates hepatic autophagy, and alleviates oxidative stress and inflammation	Preclinical	Fan et al. (2024)
	Astragaloside IV	KAT2A/PGAM1	Downregulates KAT2A expression and inhibits glycolysis	Preclinical	Zhu and Lu (2024)
	Quercetin	SIRT5/IDH2	Activates SIRT5, promotes IDH2 desuccinylation, and maintains mitochondrial homeostasis	Preclinical	Chang et al. (2024), Chang et al. (2021)
	Resveratrol	SIRT5/IDH2	Directly activates SIRT5 and alleviates oxidative stress	Preclinical	Yang et al. (2025e), Zhang L. et al. (2025)
	Puerarin	SIRT5/ALDH2	Upregulates SIRT5 expression and enhances ALDH2 detoxification function	Preclinical	Yu et al. (2024)
Multi-Target Modulators	Allicin/DATs (Diallyl Trisulfide)	SIRT5/UCP1; CPT1A/DLD	Biphasic regulation: Modulates SIRT5-UCP1 axis to improve lipid metabolism; induces cuproptosis in HSCs via CPT1A-DLD axis	Preclinical	Tian et al. (2025), Liu et al. (2023), Li R. et al. (2022), Zhang et al. (2020)
	Curcumin	SIRT5/ACSL4; CPT1A	Multi-pathway protection: Inhibits ferroptosis via SIRT5-ACSL4 axis and modulates lipid metabolism by suppressing CPT1A	Preclinical	Yashmi et al. (2024), Li W. et al. (2022), Xu et al. (2025)
TCM Formulations	Tianyang Wan (Tianyang Pill)	PKM2,ALDOA, LDHA succinylation	Rreduces succinylation of key glycolytic enzymes (PKM2, ALDOA, LDHA), inhibiting glycolysis and tumor proliferation	Translation Guided by Clinical Practice	Lu (2023)
	Huanglian Wendan Tang (Huanglian Wendan Decoction)	CPT1A/PPARα pathway	Improves lipid metabolism and alleviates NAFLD progression	Translation Guided by Clinical Practice	Zhu J. et al. (2025)
	Yiqu Gubao Wan (Yiqu Gubao Pills)	SIRT5/Mitochondria	Upregulates SIRT5 expression, enhancing mitochondrial function and coenzyme synthesis	Translation Guided by Clinical Practice	Meng (2021)

The mechanisms of action of these compound formulations indicate that TCM may, through a multi-component synergistic regulatory network, systematically modulate key catalytic enzymes and substrate proteins involved in succinylation, thereby restoring metabolic homeostasis more comprehensively. This provides new research directions and material foundations for developing liver disease treatment strategies based on network modulation rather than single-target inhibition.

### 4.5 Challenges in targeted therapy and optimization of delivery strategies

Despite their therapeutic promise, strategies targeting succinylation for liver disease treatment face significant translational hurdles. A primary challenge lies in achieving tissue- and cell-type-specific targeting, given the ubiquitous expression of succinylation machinery across organs. Systemic modulation risks disrupting physiological processes in non-hepatic tissues. For example, while inhibiting CPT1A may ameliorate hepatic steatosis, its broad expression in kidney, pancreas, and muscle could perturb systemic energy homeostasis (Liang, 2023). Further complicating clinical translation are narrow therapeutic windows exhibited by certain modulators. Allicin, for instance, displays a biphasic effect on SIRT5 activity; suboptimal dosing may shift outcomes from beneficial metabolic modulation to pathological disturbance (Zhang et al., 2020). Such dose-sensitive responses pose considerable challenges for clinical regimen design. Moreover, many promising natural bioactive compounds, such as quercetin and resveratrol, are hampered by unfavorable pharmacokinetic properties, including poor aqueous solubility, rapid metabolism, and low oral bioavailability. These limitations often prevent the attainment of effective drug concentrations in target tissues (Radeva and Yoncheva, 2025; Liu R. et al., 2025).

To address these barriers, advanced delivery platforms have emerged as enabling technologies. Ligand-functionalized nanocarriers enable cell-selective drug delivery to hepatocytes or hepatic stellate cells (Che et al., 2023; Jiao et al., 2025). Exosome-based systems exploit endogenous tropism for efficient siRNA delivery to specific cell populations (Xie et al., 2024). Mitochondria-directed carriers, such as those conjugated with triphenylphosphonium (TPP) or dendritic lipopeptides (DLP), significantly enhance intramitochondrial drug accumulation (Jiang et al., 2021; Chen et al., 2021). Additionally, metal-organic frameworks offer controlled release profiles through tunable porosity and surface chemistry (Liu et al., 2024d). Collectively, these innovative delivery strategies not only enhance drug bioavailability and targeting precision but also mitigate off-target liabilities, thereby providing critical support for the clinical advancement of succinylation-targeted therapeutics.

## 5 Discussion

### 5.1 Current research summary and core value of succinylation

This review highlights the central role of protein succinylation in the pathogenesis of liver diseases, positioning it as a critical molecular nexus linking metabolic dysfunction, epigenetic regulation, and immune microenvironment imbalances. Although significant advances have been made in tumor biology, research on succinylation in liver pathology remains nascent. The marked sensitivity of this modification to metabolic flux and its broad capacity to regulate protein function highlights its unique research value and vast unexplored potential within the liver, which is a central metabolic organ. Thus, succinylation represents not only a potential source for developing biomarkers for liver diseases but also an important extension of therapeutic targets with significant translational medical implications.

### 5.2 Key scientific questions and technical bottlenecks

Current research faces multiple technical bottlenecks that constrain a systematic understanding of the functional role of succinylation in liver diseases. The primary bottlenecks lie in detection and validation technologies. While advances in mass spectrometry and omics have expanded site identification, key challenges persist, including insufficient antibody specificity, analytical artifacts (e.g., neutral loss hindering site localization), a lack of robust methods for quantifying *in vivo* dynamics, and the loss of cell-type-specific information in bulk analyses (Azevedo et al., 2022; Hermann et al., 2022). These constraints collectively limit the functional validation of identified sites.

Beyond these technical issues, conceptual and systematic gaps remain. Mechanistic studies frequently focus on single targets, neglecting the exploration of synergistic or antagonistic networks and the crosstalk with other PTMs (Hassanzadeh et al., 2024). Furthermore, research has predominantly centered on hepatocytes, leaving the succinylation landscapes and functions in non-parenchymal cells (e.g., HSCs and Kupffer cells) poorly characterized.

To advance the field, developing detection tools with higher specificity and spatial resolution is imperative. A paradigm shift toward systems biology, integrating multi-omics and multi-cell-type analyses, is required to construct dynamic regulatory networks and fully elucidate the role of succinylation in liver physiology and disease.

### 5.3 Barriers and strategic considerations for clinical translation

Translating succinylation-targeting strategies into clinical practice presents several challenges. Although various small-molecule modulators have shown preclinical promise, their efficacy and safety lack robust support from clinical trials. It is important to note that most preclinical data are derived from rodent models, primarily mice. Significant species differences exist between mice and humans in terms of liver metabolism, immune responses, and potentially the substrate specificity or expression patterns of key succinylation enzymes (e.g., SIRT5, CPT1A) (Luo et al., 2017; Emmanuel et al., 2024). These differences may limit the direct extrapolation of therapeutic efficacy and optimal dosing from mouse models to human patients, underscoring the need for cautious interpretation of preclinical results.

Three core obstacles exist at the trial design and implementation levels: patient stratification must integrate succinylation profiles with molecular pathological features; intervention timing needs to align with disease dynamics; and efficacy evaluation requires the establishment of modification-specific biomarker systems. Furthermore, research on multi-component, multi-target TCM formulas is complicated by their unclear mechanisms of action, ambiguous active constituents, and complex pharmacodynamic foundations. To advance clinical translation in this field, a precision disease-classification system based on succinylation signatures must be established and validated through large-scale prospective cohorts for its diagnostic and prognostic value while simultaneously exploring modification-guided personalized therapies to open new avenues for the precise management of liver diseases.

### 5.4 Future research directions and breakthrough pathways

Considering the current research status and challenges, future studies should achieve systematic breakthroughs across these three dimensions. Technologically, spatially resolved single-cell multi-omics technologies should be developed to enable cell-type-specific analysis of succinylation within complex hepatic microenvironments, coupled with gene-editing tools to establish high-throughput platforms for site-specific functional annotation. Mechanistically, studies must move beyond single modifications to elucidate the crosstalk between succinylation and other PTMs (e.g., acetylation and phosphorylation), thereby revealing their central roles in metabolic–epigenetic–immune networks and constructing dynamic regulatory maps. Clinically, molecular disease subtyping systems based on succinylation profiles should be advanced, modification-specific imaging probes and liquid biopsy technologies should be developed, and temporal intervention strategies targeting dynamic modification signatures should be designed to achieve personalized precision medicine.

## 6 Conclusion

This review systematically elucidates the core regulatory mechanisms of succinylation in liver disease and evaluates its potential as a therapeutic target. Succinylation forms a dynamic metabolic–epigenetic–immune interaction network whose functional imbalance extensively participates in and drives hepatic pathological processes. In NAFLD/MASLD, succinylation exacerbates lipid accumulation by regulating lipid metabolism enzymes (e.g., CPT2 and ECHA) and the transcription factor SREBP1. In viral hepatitis, it modulates viral transcription and immune responses via histone modifications (H3K79/H3K122). During liver fibrosis, succinylation mediates HSC activation and metabolic reprogramming (e.g., PKM2). HCC drives tumor metabolic reprogramming, immune evasion, and stemness maintenance through multiple substrates (e.g., PGAM1, LACTB, and OXCT1). In liver failure, succinylation promotes hepatocyte death by impairing mitochondrial function (e.g., ALDH2) and amplifying inflammatory responses.

Based on these mechanisms, intervention strategies that target succinylation establish a multi-layered therapeutic framework that includes drug repurposing, synthetic small-molecule modulators, natural products, and TCM formulations, thereby collectively validating its feasibility as a therapeutic target. However, clinical translation encounters challenges, such as off-target risks stemming from the complexity of the modification system and the suboptimal pharmacokinetics of many candidate compounds. Future efforts should integrate multidisciplinary approaches to develop organelle-specific dynamic detection and delivery technologies, systematically decipher the crosstalk between succinylation and other PTMs, and advance modification-based disease subtyping along with prospective clinical validation.

In summary, succinylation research is undergoing a pivotal transition from mechanistic discovery to targeted intervention. By deepening our understanding of the regulatory network mechanisms, innovating precise targeting strategies, and overcoming translational barriers, this field promises to yield novel biomarkers, targets, and therapeutic approaches for the diagnosis and treatment of liver diseases, ultimately bridging the gap between basic science and clinical practice.

## Glossary

3-sucCA: 3-Succinoylcholic acid
α-KGDHC: α-ketoglutarate dehydrogenase complex
ACOX1: Acyl-CoA oxidase 1
ACSL4: Acyl-CoA synthetase long-chain family member 4
ALDH2: Aldehyde dehydrogenase 2
ALDOA: Fructose bisphosphate aldolase A
cccDNA: Covalently closed circular DNA
CPT1A: Carnitine palmitoyltransferase 1A
DATs: Diallyl trisulfide
DLD: Dihydrolipoyl dehydrogenase
DLP: Dendritic lipopeptides
DUSP1: Dual-specificity phosphatase 1
ECHA: Trifunctional enzyme subunit alpha
FXR: Farnesoid X receptor
GABA: Gamma-aminobutyric acid
H3K4me3: Histone H3 lysine 4 trimethylation
HBV: Hepatitis B virus
HBc: HBV core protein
HCC: Hepatocellular carcinoma
HIF-1α: Hypoxia-inducible factor-1α
HK1: Hexokinase 1
HSC: Hepatic stellate cells
IDH2: Isocitrate dehydrogenase 2
KAT2A: Lysine acetyltransferase 2A
KAT8: Lysine acetyltransferase 8
KMT5A: Lysine methyltransferase 5A
LACTB: Serine β-lactamase-like protein
LCAD: Long-chain acyl-CoA dehydrogenase
LDHB: Lactate dehydrogenase B
MASLD: Metabolic dysfunction-associated steatotic liver disease
Mep50: Methylosome protein 50
MFF: Mitochondrial fission factor
MOF: Metal-organic frameworks
NAFLD: Non-alcoholic fatty liver disease
OXCT1: Oxoacid CoA-transferase 1
PCK1: Phosphoenolpyruvate carboxykinase 1
PGAM1: Phosphoglycerate mutase 1
PKM2: Pyruvate kinase M2
PPP2R1A: Protein phosphatase 2 scaffold subunit alpha
PRMT5: Protein arginine methyltransferase 5
PTK2: Protein tyrosine kinase 2
PTM: Post-translational modifications
ROS: Reactive oxygen species
SERCA2a: Sarcoplasmic/endoplasmic reticulum calcium ATPase 2a
STAT1: Signal transducer and activator of transcription 1
SREBP1a: Sterol regulatory element-binding protein 1a
STUB1: STIP1 homology and U-box containing protein 1
SUCLA2: Succinyl-CoA ligase (ADP-forming) subunit beta
SucCoA: Succinyl-coenzyme A
TACE: Transarterial chemoembolization
TBK1: TANK-binding kinase 1
TCA: Tricarboxylic acid
TCM: Traditional Chinese Medicine
TPP: Triphenylphosphonium
TSG: 2,3,5,4′-Tetrahydroxystilbene-2-O-β-D-glucoside
UCP1: Uncoupling protein-1
